# ASSOCIATION OF SLEEP MEASURES WITH IMMUNE CELL PHENOTYPES IN THE FRAMINGHAM HEART STUDY

**DOI:** 10.1093/geroni/igae098.3515

**Published:** 2024-12-31

**Authors:** Ahmed Ragab, Jiachen Chen, Yumeng Cao, Margaret Doyle, Kathryn Lunetta, Joanne Murabito

**Affiliations:** Boston University, Boston, Massachusetts, United States; Boston University School of Public Health, Boston, Massachusetts, United States; Boston University, Boston, Massachusetts, United States; University of Vermont Larner College of Medicine, Colchester, Vermont, United States; Boston University, Boston, Massachusetts, United States; Chobanian and Avedisian School of Medicine, Boston University, Boston, Massachusetts, United States

## Abstract

Sleep plays a significant role in regulating immune functions. Disordered sleep is linked to higher risk of infection and chronic inflammation. The relationship between sleep and the immune system is reciprocal, with each influencing the other. Adequate sleep is crucial for maintaining a balanced immune response and overall health. The associations between sleep measures and comprehensive immune cell phenotypes have not been well studied. Among Framingham Heart Study (FHS) Offspring participants who participated in the Sleep Study (1996-1998) and had immune cell data, we investigated five different sleep measures, average weighted sleeping hours per week, apnea hypopnea index with 3% oxygen desaturation, self-reported snoring >3 times a week, percentage of sleep time with oxygen saturation below 90% (TST90%) and the Epworth Sleepiness Scale (ESS) in relation to fourteen immune cell phenotypes. At exam 7 (1998-2001) using flow cytometry immune cells were profiled including T cells, B cells, NK cells, CD4+, CD8+, CD4+CD28-CD27-, CD8+CD28-CD27-, CD4+ TEMRA, CD8+ TEMRA, CD4+/CD8+ ratio, CD4+(TN/TM), CD8+ (TN/TM), CD8+ Tc17/Treg ratio and CD4+ Th17/Treg ratio. Linear Mixed Effects models were used for analysis with adjustment for covariates. Among 199 participants (mean age 62 years, range 42 to 83 years, 51% female), CD8+ TEMRA and CD8+CD28-CD27- were significantly associated with ESS and TST90% after adjusting for age, sex, CMV status, time difference between exams, family relationships, sleep medications and cardiovascular risk factors (Beta Coeffs:0.05, -0.02; FDRs=0.03, 0.03) respectively. We conclude that CD8+ TEMRA and CD8+CD28-CD27- are associated with daytime sleepiness and sleep-related hypoxemia. Further investigation is justified.

